# Case Report: An Unusual First Manifestation of a Pheochromocytoma

**DOI:** 10.3389/fendo.2021.697202

**Published:** 2021-07-07

**Authors:** Qiangrong Zhai, Ci Tian, Zhengzhao Deng, Congrong Liu, Qingbian Ma

**Affiliations:** ^1^ Department of Emergency, Peking University Third Hospital, Beijing, China; ^2^ Department of Endocrinology, Peking University Third Hospital, Beijing, China; ^3^ Department of Pathology, Peking University Third Hospital, Beijing, China

**Keywords:** pheochromocytoma, diffuse alveolar hemorrhage, case report, hemoptysis, first manifestation

## Abstract

We present a 30-year old male presented with hemoptysis as a first manifestation and pulmonary CT scan characteristic of diffuse alveolar hemorrhage. Further abdominal examination discovered a left adrenal mass. Elevated catecholamine and metanephrine levels and subsequent adrenalectomy confirmed the diagnosis of pheochromocytoma. Symptoms of pheochromocytoma are highly variable, which could potentially delay the diagnosis. With careful investigation, emergency medicine practitioners need to be aware of the cunning and changeful manifestations in pheochromocytoma.

## Introduction

Pheochromocytoma is not rare for emergency medicine practitioners. It can confound clinicians with more than 30 medical disorders (1). Identifying the rare symptom of pheochromocytoma is a challenge for physicians (2). Most cases presented are caused by the excess concentrations of circulating catecholamines. Episodic symptoms may occur in spells or paroxysms that can be extremely variable in presentation but typically include forceful heartbeat, pallor, tremor, headache, and diaphoresis (3). Meanwhile, rare performance should be noted as well. Here, we present a rare case of pheochromocytoma presented as diffuse alveolar hemorrhage.

## Case Report

A 30-year old male presented to the emergency department complained of hemoptysis as a first manifestation a year ago without obvious predisposing cause. The hemoptysis was manifested as bloody sputum, with a total volume of 50–100 ml. The accompanying symptoms included paroxysmal chest distress, shortness of breath, and palpitation. During his stay in the emergency department, the physical examination was unremarkable. The pulmonary CT (taken in another hospital) showed mild infection. The abdominal ultrasound and echocardiography were both normal. The reexamination of pulmonary CT showed normal. The diagnosis of pneumonia was suspected then. His symptoms were relieved quickly after hemostatic therapy and antibiotic treatment. The patient was discharged without further evaluation. After a year’s duration, those paroxysmal symptoms sometimes start with nausea and emesis. This time, he visited the emergency department complaining of a recent attack. He was well before, without a history of medication. Physical examination was unremarkable. He was conscious, afebrile, and oriented, with a blood pressure of 107/85 mmHg, a heart rate of 79/min, and a respiratory rate of 18 breaths/min.

A complete blood count revealed the following: white blood cell 23,720/mm**^3^**, hemoglobin 18.1 g/dl, hematocrit 52%, and platelets 275,000/mm**^3^**. Liver function test results were ALT 62 IU/L, AST 58 IU/L, and LDH 326 IU/L. His blood glucose was 297 mg/dl, and serum creatinine was 114 ummol/L. Prothrombin time and activated partial thromboplastin time were 10.7 and 29.2 s, respectively. The examination of the urine showed proteinuria and glucosuria.

**Figure 1:**
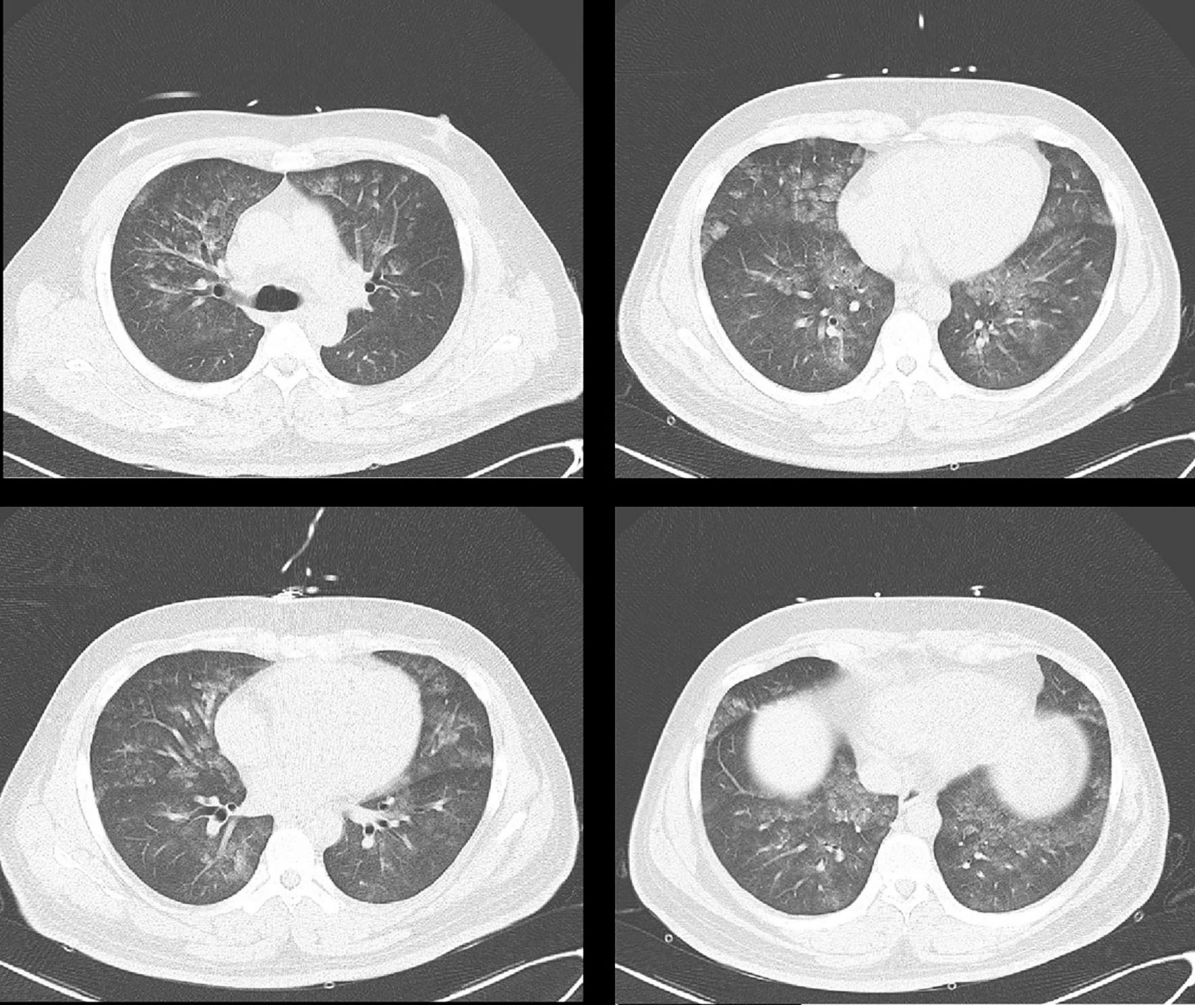
Diffuse lesions in both lungs, showing patchy high-density shadow and ground-glass opacity.

At admission, the pulmonary CT scan showed patchy high-density shadow and ground-glass opacity, compatible with diffuse alveolar hemorrhage (DAH) (see **Figure 1**). The bronchoscopy confirmed the diagnosis. The bronchoalveolar lavage reported macrophage (86%), lymphocyte (1.5%), neutrophil (12.5%). Autoimmune diseases were suspected at first. Further laboratory tests showed anti-DNA (anti-double stranded DNA), c-ANCA (c-anti-neutrophil cytoplasmic antibodies), anti-MPO (anti-myeloperoxidase antibody), ANA (anti-nuclear antibody), and anti-GBM (anti-glomerular basement membrane) antibodies were negative. Blood and sputum cultures were negative as well. The cortisol levels were 2.5 µg/dl (0 AM), 12.3 µg/dl (8 AM, reference value 5–25 µg/dl), 11.6 µg/dl (4 PM, reference value 2.5–12.5 µg/dl). Moreover, CT reexamination showed the diffuse alveolar hemorrhage resolved completely 5 days after the initial CT without any targeted treatment.

Acute coronary syndrome and stress-induced cardiomyopathy were both suspected. However, the patient did not show any signs of pulmonary edema. Biomarkers of myocardial injury were normal at admission. The echocardiography was normal with favorable systolic and diastolic functions. The electrocardiogram revealed normal sinus rhythm. The blood pressure remained normal under close monitoring.

**Figure 2:**
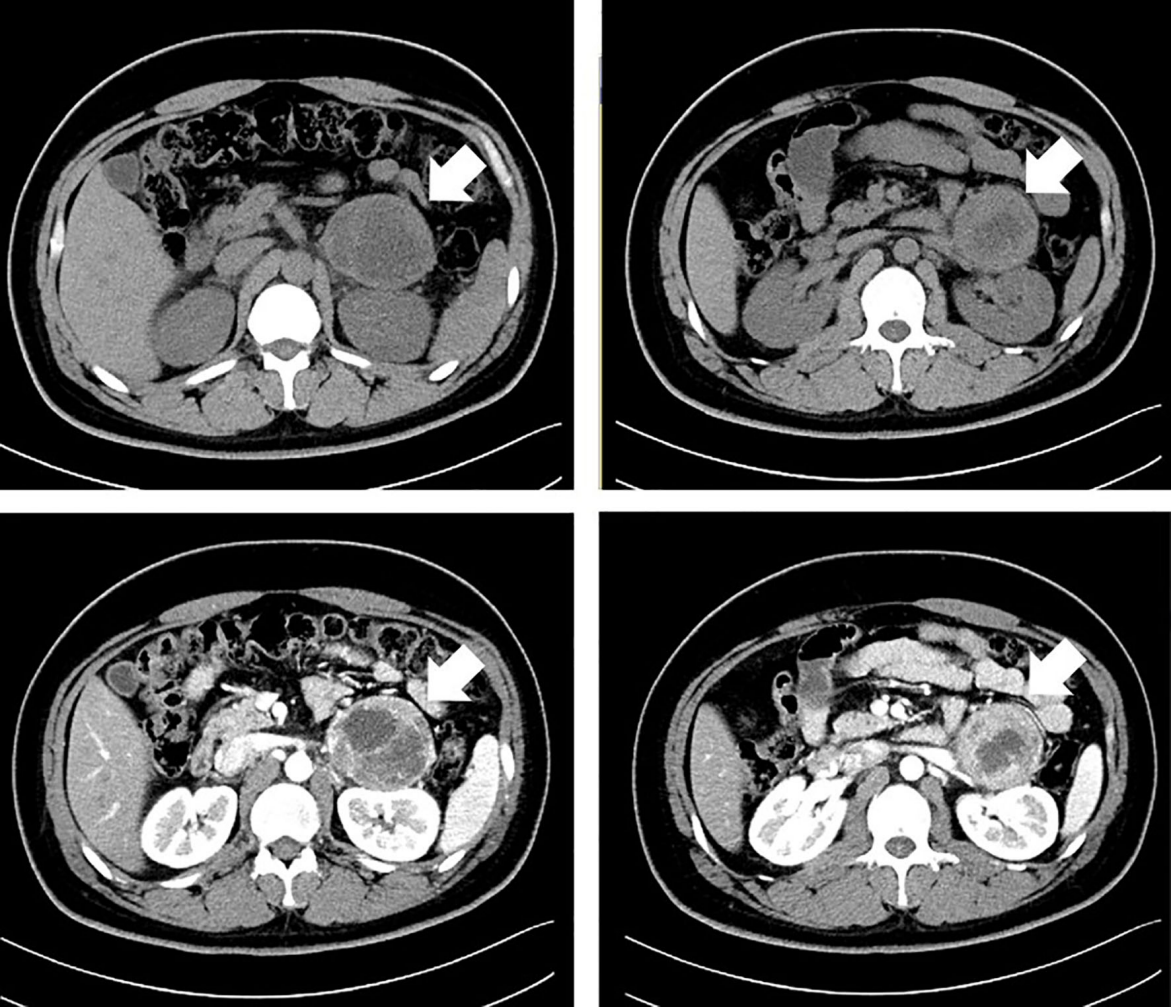
A computed tomographic (CT) scan of the abdomen with intravenous contrast in the patient with a left adrenal mass.

The paroxysmal symptoms indicated high levels of catecholamine. The abdominal CT scan discovered left adrenal mass with intravenous contrast (69 * 61 * 62 mm) (see **Figure 2**). The diagnosis of pheochromocytoma was confirmed biochemically by the elevated catecholamine and metanephrine levels in the blood [metanephrine 2,506.2 pmol/L (<605.9 pmol/L), normetanephrine 16,279.2 pmol/L (413.9–4434.2 pmol/L)], and urine (VMA 40.3 µg/24 h, reference value 1.9–13.6 µg/24 h).

**Figure 3:**
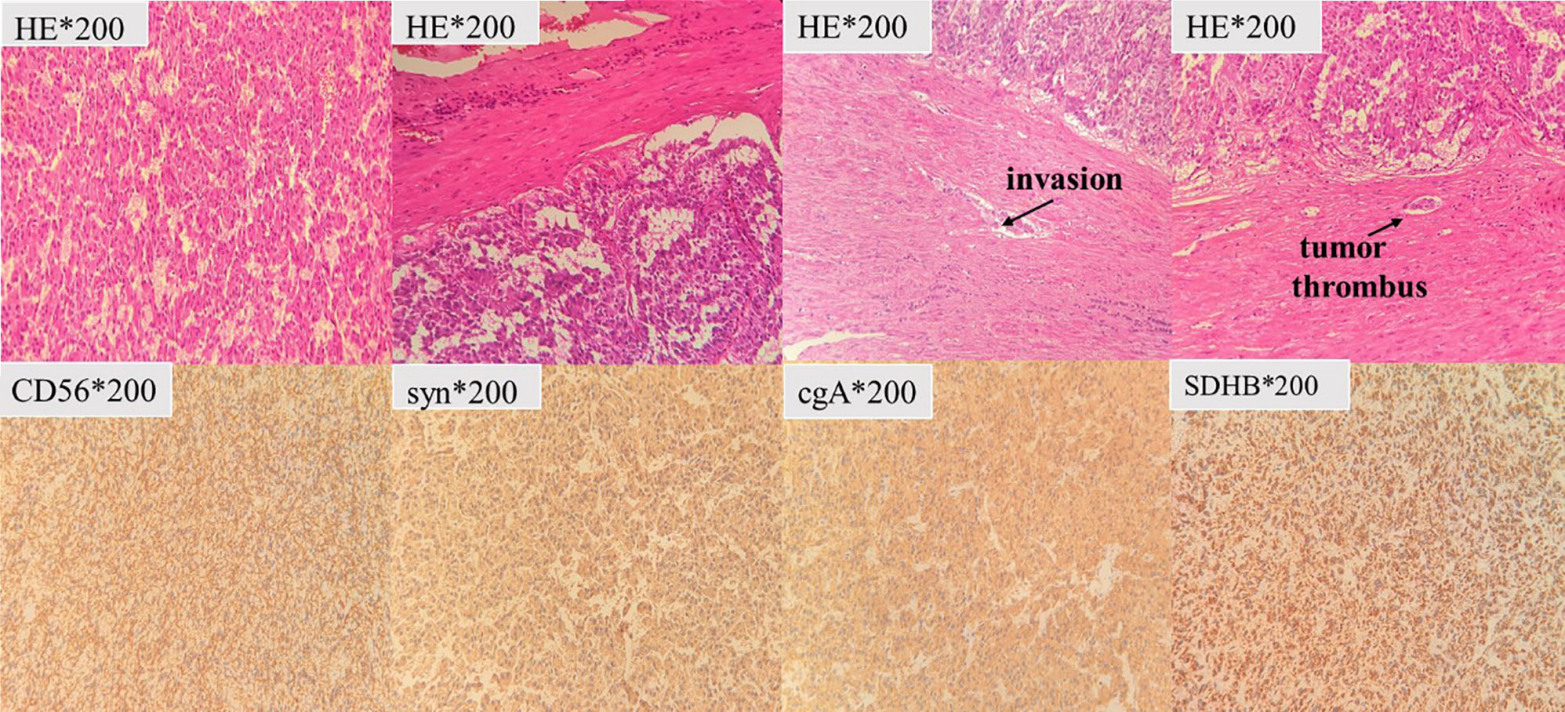
Tumor cells are diffusely distributed, with beam-like structures and capsular invasion. CD56, syn and cgA expression is positive, supporting the diagnosis of pheochromocytoma.

The diagnosis was considered as “pheochromocytoma, diffuse alveolar hemorrhage”. The patient received treatment of phenoxybenzamine, doxazosin mesylate, and moxifloxacin. During the hospital stay, the patient’s condition was getting better without any symptoms. Two months after discharge from the respiratory intensive care unit, the patient revisited the urology surgery department. The left adrenalectomy was performed by laparoscopy. Pathology examination revealed pheochromocytoma with moderate atypia histological features, immunohistochemical staining [Syn (+), SDHB (+), CD56(+), Ki-67(−)] (see **Figure 3**).

We followed the patient for 6 months. His symptoms gradually improved, and he was discharged.

## Discussions

A broad spectrum of potential presenting symptoms of pheochromocytoma includes the classic triad of headaches, palpitations, and profuse sweating (4). The immediate cause of the patient’s visit to the emergency department was hemoptysis, which is rare. In 1986, a report described hemoptysis as a presenting symptom of pheochromocytoma in a patient with adrenal pheochromocytoma (5). Over the years, only a few published articles mentioned resembling symptoms, of which only one case explicitly identified the cause as with typical CT manifestation (6). It is a symptom that is overlooked and underestimated. Considering pathologic result, current thinking is that all pheochromocytomas have some metastatic potential. Putative adverse features fall into five categories: invasion, architectural variation, cytological variation, necrosis, and proliferative activity (7). Although invasion was observed in our patient, the Ki-67 proliferation index is less than 1% in our case. Further genetic counseling should be considered to predict long-term outcome.

Several mechanisms have been proposed for this diffuse alveolar hemorrhage. Kimura Y et al. (8) proposed that the increased activation of the coagulation cascade may have contributed to hemoptysis. Hemoptysis was also attributed to elevated blood pressure and pulmonary venous hypertension leading to pulmonary edema and hemorrhage (5). Those theories raised that rather than the pulmonary system itself, other organ damages caused by excessive catecholamines led to pulmonary lesion. In the lung, circulating catecholamines have two major effects. They relax bronchial smooth muscles and decrease fluid secretion from bronchial glands (9). In the pulmonary circulation, the effect is complicated. Animal model shows norepinephrine causes dose- and stimulus frequency-dependent increases in pulmonary vascular resistance. However, when pulmonary vascular tone was enhanced and a receptor is blocked, norepinephrine and nerve stimulation caused dose- and frequency-dependent decreases in pulmonary vascular resistance (10). As a result, these altered pulmonary vascular responses may effect pulmonary capillary pressure, a major determinant of lung fluid balance (11). Based on that, Bourvis N et al. (12) hypothesized that capillary permeability increases by the effect of catecholamine in pheochromocytoma. No conclusive evidence has been raised so far yet. In our case, no evidence supports coagulation dysfunction, cardiac dysfunction, or elevated blood pressure. Whether it was caused by increased capillary permeability remains unclear.

On the other hand, DAH often appears with an established associated disease or be the initial manifestation of underlying systemic disease (13). It is hard to relate pheochromocytoma as an etiology in DAH. The immediate cause of the patient’s visit to the emergency department a year ago was hemoptysis. When the patient first came to us, we did not continue to investigate the cause of hemoptysis as pulmonary CT appeared to be normal afterwards. A careful abdominal examination was ignored as we focused mainly on respiratory diseases. A lesson should be learned here. Numerous disorders can cause signs and symptoms that may prompt the clinician to test for pheochromocytoma. Although hemoptysis is a rare symptom of pheochromocytoma, the paroxysmal symptoms are typical indicators of pheochromocytoma. This case report reminds emergency medicine practitioners to be aware of the cunning and changeful manifestations in pheochromocytoma.

## Data Availability Statement

The original contributions presented in the study are included in the article. Further inquiries can be directed to the corresponding authors.

## Ethics Statement

Ethical review and approval was not required for the study on human participants in accordance with the local legislation and institutional requirements. The patients/participants provided their written informed consent to participate in this study. Written informed consent was obtained from the individual(s) for the publication of any potentially identifiable images or data included in this article.

## Author Contributions

QZ and CT contributed to the manuscript preparation. ZD contributed to the manuscript revision. CL and QM contributed to the concepts and design. All authors contributed to the article and approved the submitted version.

## Conflict of Interest

The authors declare that the research was conducted in the absence of any commercial or financial relationships that could be construed as a potential conflict of interest.
